# Supplementary material for: Coil-globule transitions drive discontinuous volume conserving deformation in locally restrained gels
Source: Nat Commun. 2018 May 25;9:2062. doi: 10.1038/s41467-018-04533-w (PMC5970185; doi:10.1038/s41467-018-04533-w)
Supplement: Supplementary file 1 — Supplementary Information [file 41467_2018_4533_MOESM1_ESM.pdf]

Supplementary Information: Coil-globule transitions drive discontinuous volume-conserving deformations in restrained gels

Yamamoto *et al*

E-mail:

*To whom correspondence should be addressed

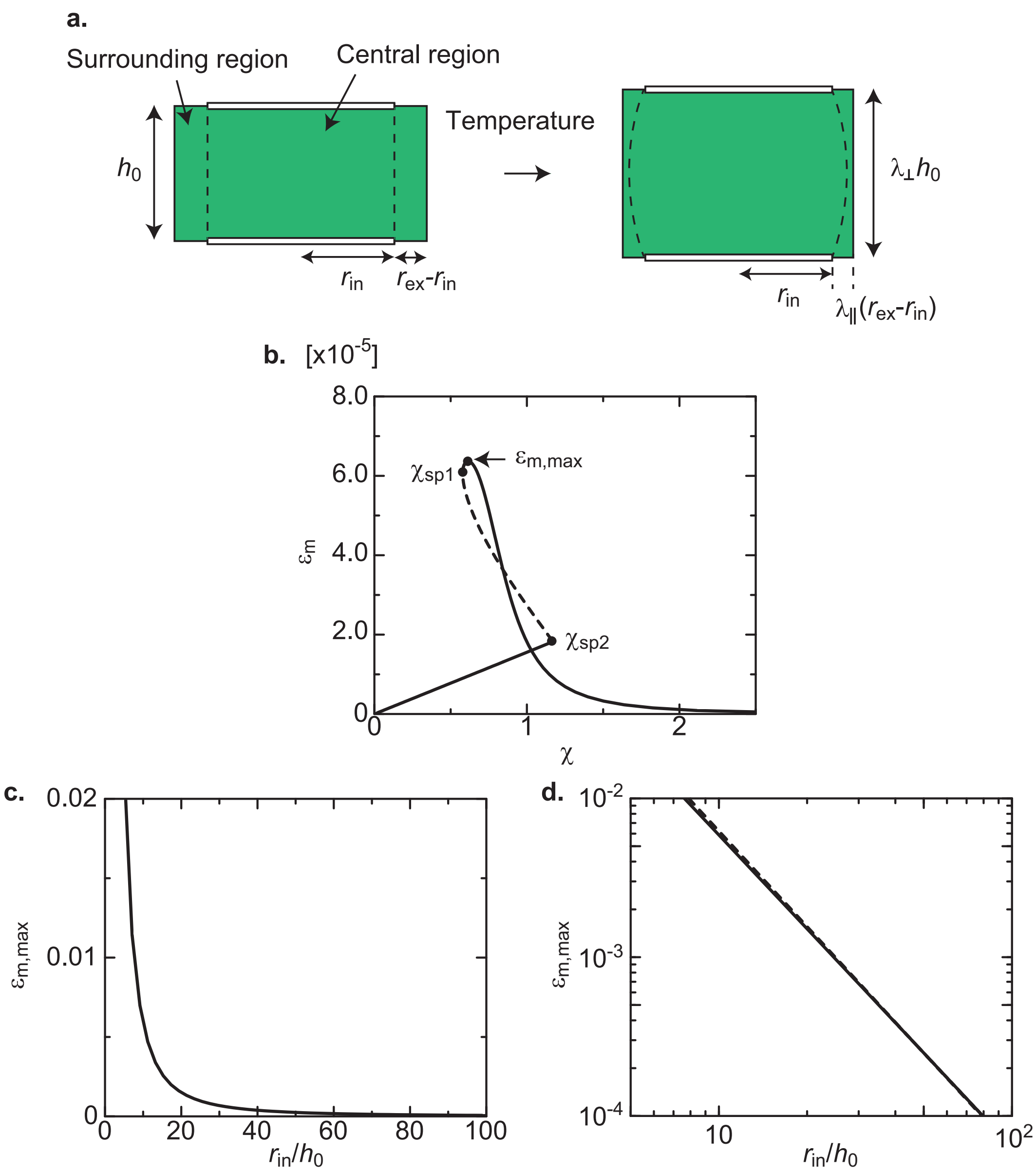

Supplementary Figure 1 Lateral deformation of the central region. a. The central region of a unit cell can show a non-uniform deformation in the lateral direction when one changes the interaction parameter χ. The amplitude ϵ_m of the deformation is defined by Supplementary Equation (47). **b.** The amplitude ϵ_m of the deformation is shown as a function of the interaction parameter χ for the aspect ratio r_{in}/h_0 of the unit cell is 100. The stable solutions are shown by solid curves and the unstable solution is shown by a broken curve. The unit cell shows the maximum deformation $\epsilon_{m,max}$ at an interaction parameter. **c.** The maximum deformation $\epsilon_{m,max}$ is shown as a function of the aspect ratio r_{in}/h_0 of the unit cell. This function is shown by a log-log plot in **d**. The broken curve in **d** scales as $(r_{in}/h_0)^{-2}$. **b**, **c**, and **d** are derived by minimizing Supplementary Equation (46) with respect to $\lambda_\perp$ and ϵ_m for $g_0 = 1.0 \times 10^{-3}$ and $s = 0.2$ (see Supplementary Equations (11) and (22) for the definition).

a.

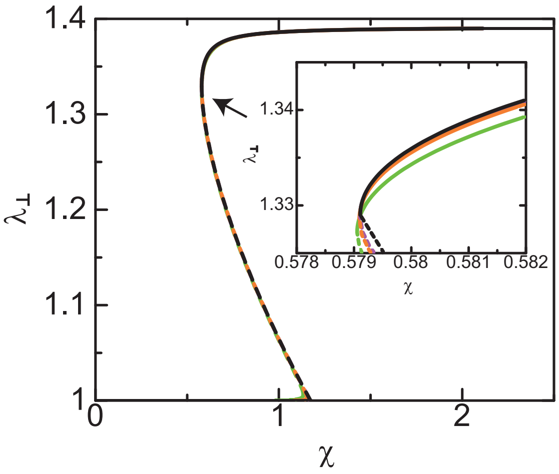

b.

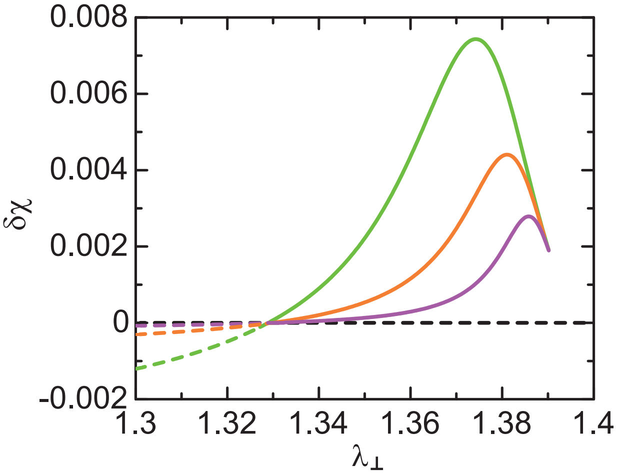

Supplementary Figure 2 Extension ratio $\lambda_\perp$ as a function of the interaction parameter χ with the lateral deformation in the central region. a. The extension ratio $\lambda_\perp$ in the normal direction is calculated as a function of the interaction parameter χ for cases in which the central region shows lateral deformation (see Supplementary Note 4). The solid curves show the stable solutions and the broken curve shows the unstable solution. The black curve is the case in which the central region does not show the lateral deformation. The region near the unstable point χ_{sp1} of the deformed state is magnified in the inset. **b.** The deviation $\delta\chi$ of the interaction parameter χ due to the lateral deformation of the central region is shown as a function of the extension ratio $\lambda_\perp$. The values of the aspect ratio r_{in}/h_0 used for the calculations are 25.0 (light green), 50.0 (orange), and 100.0 (purple). We used $g_0 = 1.0 \times 10^{-3}$ and $s = 0.2$ for the calculations (see Supplementary Equations (11) and (22) for the definition).

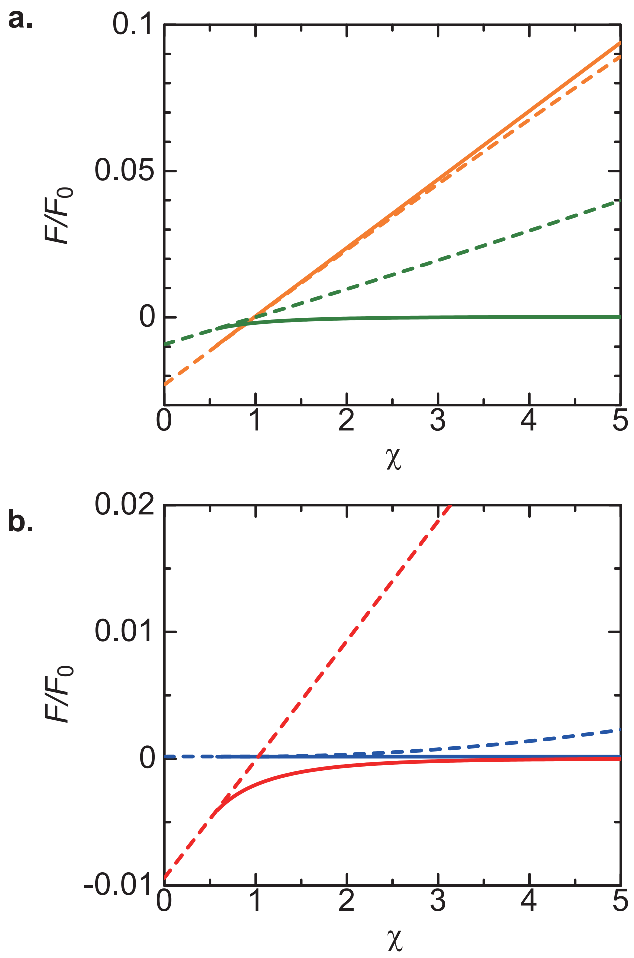

Supplementary Figure 3 Free energy is broken down to components. a. The free energy components of the central region (orange) and surrounding region (green) are shown as functions of the interaction parameter χ. The free energy of the deformed state (the solution at $\lambda_\perp > 1$) is shown by the solid curves and the free energy of the undeformed state (the solution at $\lambda_\perp = 1$) is shown by the broken curves. **b.** The elastic free energy (red) and the mixing free energy (blue) of the surrounding region are shown as functions of the interaction parameter χ. The free energy of the deformed state (the solution at $\lambda_\perp > 1$) is shown by the solid curves and the free energy of the undeformed state (the solution at $\lambda_\perp = 1$) is shown by the broken curves. These curves are calculated for the volume ratio $s = 0.2$ and rescaled shear modulus $g_0 = 1.0 \times 10^{-3}$. The free energy components are rescaled by F_0 ($\equiv k_{\rm B}T\pi r_{\rm in}^2 h_0/v_0$).

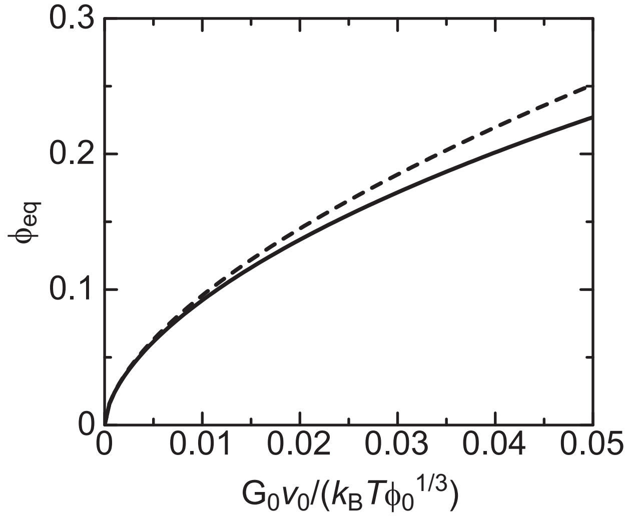

Supplementary Figure 4 Volume fraction after the swelling process. The volume fraction $\phi_{\rm eq}$ after the swelling process is shown as a function of the rescaled shear modulus g_0 ($\equiv G_0 v_0/(k_{\rm B} T \phi_0^{1/3})$) of the polymer network (solid curve). The volume fraction $\phi_{\rm eq}$ has an asymptotic form $\phi_{\rm eq} = (2g_0)^{3/5}$ for small values of the rescaled shear modulus g_0 (broken curve).

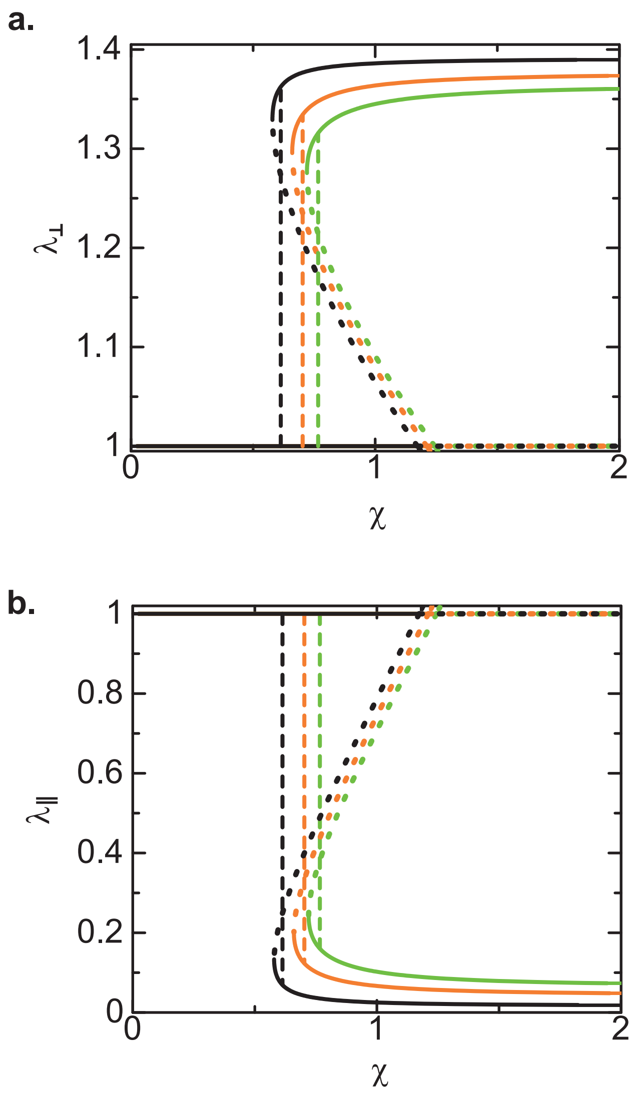

Supplementary Figure 5 Dependence of the deformation of the composite gel on the shear modulus of the polymer network. The extension ratios, $\lambda_\perp$ (**a**) and $\lambda_\parallel$ (**b**), (defined by fig. 1 in the main article) are shown as a function of the interaction parameter χ. We used $s = 0.2$ for the value of the volume ratio (defined by Supplementary Equation (22)) and $g_0 = 1.0 \times 10^{-3}$ (black), 5.0×10^{-3} (orange), and 1.0×10^{-2} (light green) for the values of the rescaled shear modulus g_0 of the polymer network (defined by Supplementary Equation (11)).

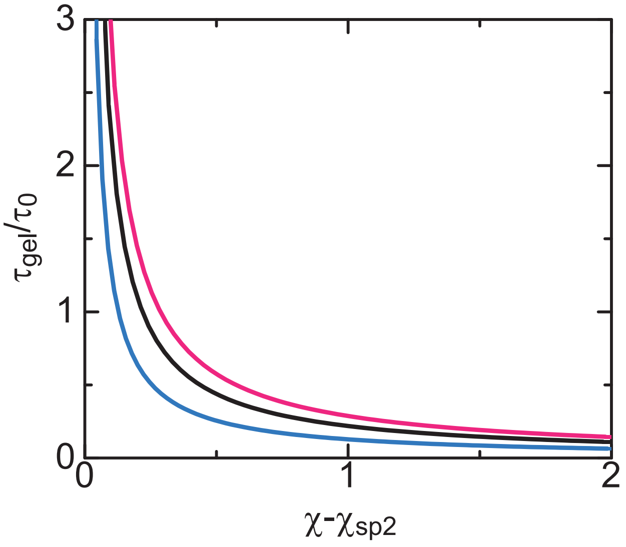

Supplementary Figure 6 Time scale of the deformation. The rescaled response time τ_{gel}/τ_0 $(= \alpha_0^{-1})$ of the deformation (in the short time scale) is shown as a function of the interaction parameter χ (subtracted by the second threshold value χ_{sp2} at which the undeformed state becomes unstable). The values of the volume ratio s (defined by Supplementary Equation (22) and equation (13) in the main article) used for the calculations are 0.1 (cyan), 0.2 (black), and 0.3 (magenta). The rescaled shear modulus g_0 (defined by Supplementary Equation (11) and equation (9) in the main article) is fixed to 1.0×10^{-3}.

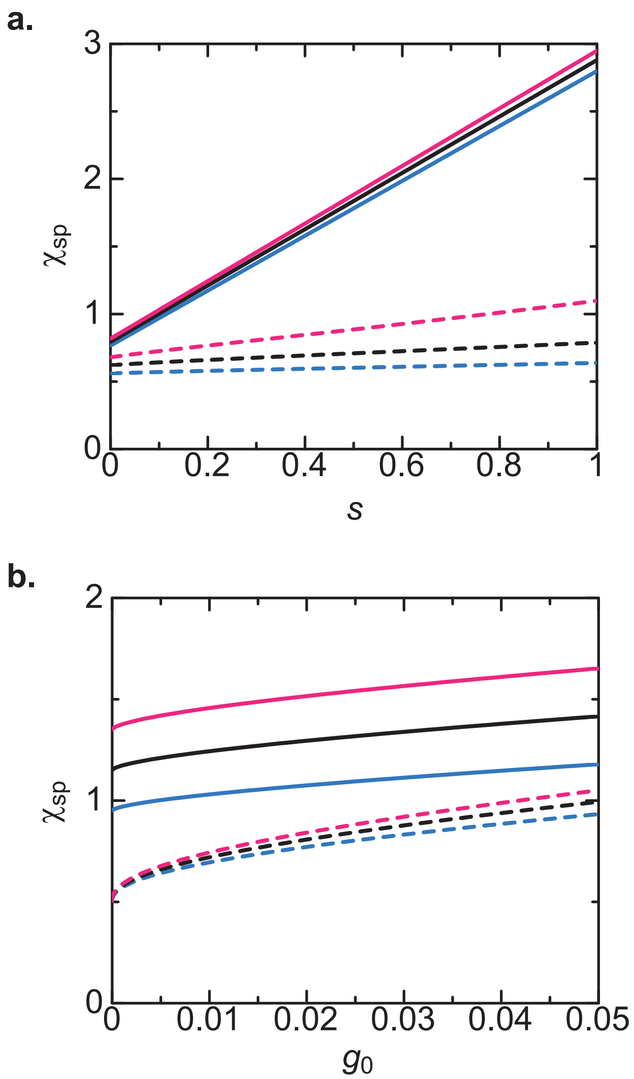

Supplementary Figure 7 Threshold values of interaction parameter χ. The threshold values, $\chi_{\rm sp1}$ and $\chi_{\rm sp2}$, of the interaction parameter are shown as functions of (**a**) the volume ratio s and (**b**) the rescaled shear modulus g_0 (defined by Supplementary Equation (11) and equation (9) in the main article). The first threshold values $\chi_{\rm sp1}$ (at which the deformed state becomes unstable) are shown by broken curves and the second threshold values $\chi_{\rm sp2}$ (at which the undeformed state becomes unstable) are shown by solid curves. The values of the rescaled shear modulus g_0 used in the calculations in **a** are 1.0×10^{-3} (cyan), 5.0×10^{-3} (black), and 1.0×10^{-2} (magenta). The values of the volume ratio s used for the calculations in **b** are $s = 0.1$ (cyan), 0.2 (black), and 0.3 (magenta).

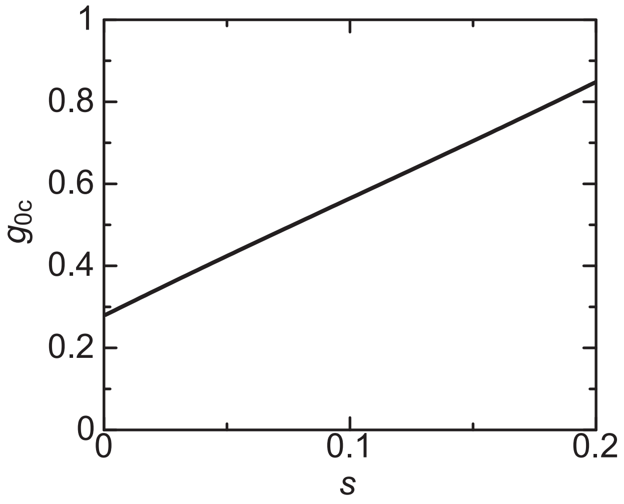

Supplementary Figure 8 The critical rescaled shear modulus g_{0c} vs the volume ratio s. The critical rescaled shear modulus g_{0c} (the rescaling factor is shown in the Supplementary Equation (11) and equation (9) in the main article) is shown as a function of the volume ratio s ($\equiv (r_{ex} - r_{in})/r_{in}$).

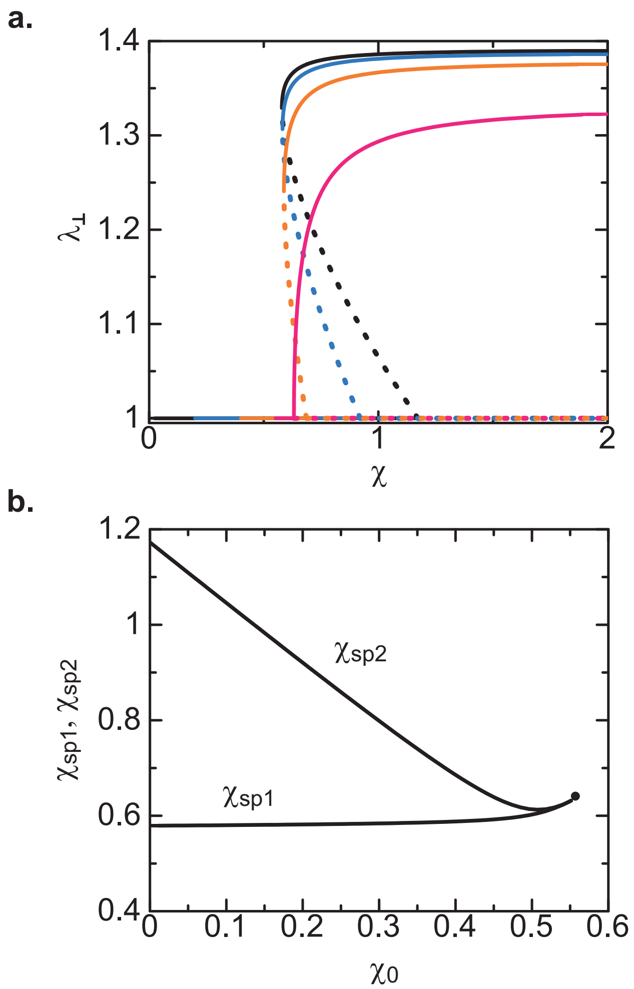

Supplementary Figure 9 The effects of the solvent quality during the swelling process on the volume conserving transitions. a. The extension ratio $\lambda_\perp$ in the normal direction is shown as a function of the interaction parameter χ for cases in which the gel is swollen in the solvent of the interaction parameter $\chi_0 = 0.0$ (black), 0.2 (cyan), 0.4 (orange), and 0.55121 (magenta) in the swelling process. **b.** The interaction parameters, at which the deformed and undeformed states become unstable, $\chi_{\rm sp1}$ and $\chi_{\rm sp2}$, (see also fig. 3 in the main article for the definition) are shown as a function of the interaction parameter χ_0 during the swelling process. We used $g_0 = 1.0 \times 10^{-3}$ and $s = 0.2$ for the calculations (see Supplementary Equations (11) and (22) for the definition of g_0 and s).

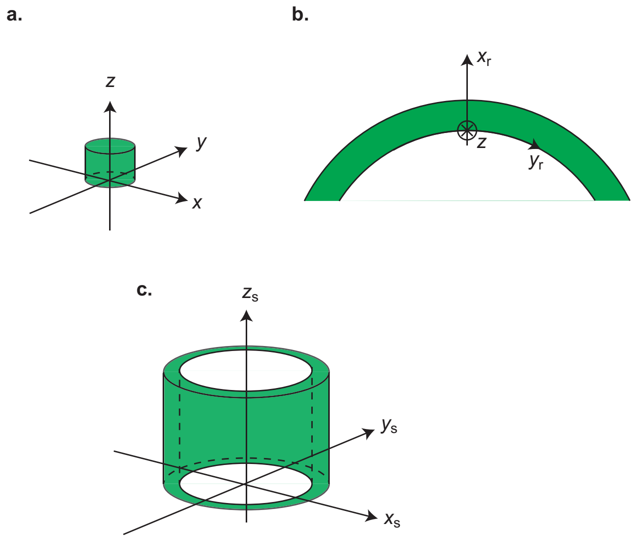

Supplementary Figure 10 Coordinate systems. a. We use the coordinate system $\mathbf{r}_0 = (x, y, z)$ to represent material points in the reference state (before the polymer network is swollen in a solvent). z is normal to the nanosheets. x and y are parallel to the nanosheets. **b.** We use the coordinate system (x_r, y_r, z) to represent the positions of material points in the surrounding region. x_r and y_r are the distance in the normal and parallel to the circumference of the nanosheet. z is normal to the nanosheet. **c.** We use the coordinate system $\mathbf{r}_\mathrm{s} = (x_\mathrm{s}, y_\mathrm{s}, z_\mathrm{s})$ to represent material points in a swollen gel. z_s is normal to the nanosheets. x_s and y_s are parallel to the nanosheets.

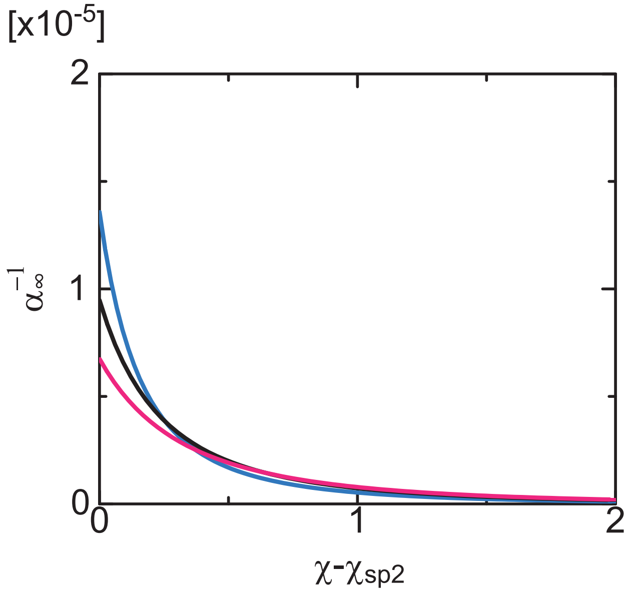

Supplementary Figure 11 Time scale of the deformation. The rescaled response time α_∞^{-1} of the deformation (in the long time scale) is shown as a function of the interaction parameter χ (subtracted by the second threshold value $\chi_{\rm sp2}$ at which the undeformed state becomes unstable). The values of the volume ratio s (defined by Supplementary Equation (22) and equation (13) in the main article) used for the calculations are 0.1 (cyan), 0.2 (black), and 0.3 (magenta). The rescaled shear modulus g_0 (defined by Supplementary Equation (11) and equation (9) in the main article) is fixed to 1.0×10^{-3}.

Supplementary Methods

We here treat a polymer network before it is swollen in a solvent as the reference state. The position of a material point in the reference state has the form

$$\mathbf{r}_0 = (x, y, z), \tag{1}$$

see the Supplementary Figure 10**a**. The material point is displaced to a position $\mathbf{r}(x, y, z)$ after the deformation. The extent of the deformation is represented by the metric tensor

$$g_{\alpha\beta} = \mathbf{r}_\alpha \cdot \mathbf{r}_\beta, \tag{2}$$

where α and β are 1, 2, or 3. $\mathbf{r}_\alpha$ is the derivative of the position $\mathbf{r}(x, y, z)$ after the deformation with respect to x_α ($x_1 = x$, $x_2 = y$, and $x_3 = z$). Here and after, we use the Einstein convention, with which repeated subscripts in the same term imply the sum over possible values of the indices (for example, $g_{\alpha\alpha} = g_{11} + g_{22} + g_{33}$).

The free energy of the gel has the form

$$F_{\text{gel}} = \int dV_0 \left[f_{\text{ela}} + \frac{\phi_0}{\phi} f_{\text{sol}} \right], \tag{3}$$

where f_{ela} is the volume density of the elastic energy and f_{sol} is the volume density of the mixing free energy. ϕ_0 is the volume fraction of the polymer network in the reference state. ϕ is the volume fraction of the polymer network after the deformation and has the form

$$\phi = \frac{\phi_0}{\sqrt{g}}, \tag{4}$$

where g is the determinant of the metric tensor.

The volume density of the elastic energy has the form

$$f_{\mathrm{ela}} = \frac{1}{2} G_0 \left(g_{\alpha\alpha} - 3 \right), \tag{5}$$

where G_0 is the shear modulus of the polymer network and $g_{\alpha\alpha}$ is the trace of the metric tensor. The volume density of the mixing free energy has the form

$$f_{\mathrm{sol}} = \frac{k_{\mathrm{B}} T}{v_0} \left[(1 - \phi) \log(1 - \phi) + \chi \phi (1 - \phi) \right], \tag{6}$$

where χ is the interaction parameter that represents the magnitudes of the attractive monomer-monomer and solvent-solvent interactions, relative to monomer-solvent interactions.[1] v_0 is the volume of a monomer. k_{B} is the Boltzmann constant and T is the absolute temperature.

Supplementary Note 1

Derivation of force balance equation in swelling process

The position of a material point after the swelling process has the form

$$\mathbf{r}_{\mathrm{s}} = \lambda_{\mathrm{eq}} (x, y, z), \tag{7}$$

where λ_{eq} is the swelling ratio. The trace and determinant of the metric tensor $g_{\alpha\beta}$ has the forms $g_{\alpha\alpha} = 3\lambda_{\mathrm{eq}}^2$ and $g = \lambda_{\mathrm{eq}}^6$. By using these forms, Supplementary Equation (3) is rewritten in the form

$$\frac{F_{\mathrm{gel}}}{\pi r_{\mathrm{ex}}^2 h_0} = \frac{3}{2} G_0 \left(\lambda_{\mathrm{eq}}^2 - 1 \right) + \frac{\phi_0}{\phi_{\mathrm{eq}}} f_{\mathrm{sol}}(\phi_{\mathrm{eq}}), \tag{8}$$

where ϕ_{eq} ($\equiv \phi_0 / \lambda_{\mathrm{eq}}^3$) is the volume fraction after the swelling process. Minimizing Supplementary Equation (8) with respect to the swelling ratio λ_{eq} leads to the force balance

equation

$$-\frac{G_0}{\lambda_{\rm eq}} + \Pi_{\rm sol}(\phi_{\rm eq}) = 0, \tag{9}$$

The osmotic pressure $\Pi_{\rm sol}(\phi)$ has the form

$$\begin{aligned} \Pi_{\rm sol}(\phi) &= \phi^2 \frac{\partial}{\partial \phi}\left(\frac{f_{\rm sol}(\phi)}{\phi}\right) \\ &= \frac{k_{\rm B}T}{v_0}\left[-\log(1-\phi) - \phi - \chi\phi^2\right], \end{aligned} \tag{10}$$

where we used Supplementary Equation (6) to derive the last form.

For simplicity, we treat cases in which the polymer network is swollen in an athermal solvent ($\chi = 0$) in the main article (however, it is straightforward to take into account the quality of the solvent used for the swelling process; it is taken into account by using Supplementary Equation (10) with $\chi = \chi_0$ ($\neq 0$) to solve Supplementary Equation (9), see Supplementary Figure 9). When the gel is swollen in an athermal solvent, the swelling ratio $\lambda_{\rm eq}$ is a function only of the rescaled shear modulus

$$g_0 = \frac{G_0 v_0}{k_{\rm B}T\phi_0^{1/3}}, \tag{11}$$

see Supplementary Figure 4. For small values of the rescaled shear modulus, the volume fraction $\phi_{\rm eq}$ has an asymptotic form

$$\phi_{\rm eq} = (2g_0)^{3/5}, \tag{12}$$

see the broken curve in Supplementary Figure 4.

Supplementary Note 2

Free energy as a function of the interaction parameter χ

We prepare a composite gel by embedding solid nanosheets in a swollen gel. The nanosheets are cofacially oriented and are aligned in a stack of layers. We simplify the composite gel is an assembly of unit cells, where each of them is composed of the central region, sandwiched by the two registered nanosheets, and the surrounding region (see fig. 1 in the main article).

We assume that the nanosheets are strongly adhered to the polymer network and restrain the deformation of the network. Because of the restraint, the central region can deform in the normal to the nanosheets (with an extension ratio $\lambda_\perp$), but cannot in the lateral directions (see also fig. 1 in the main article). The surrounding region can freely deform in the radial direction (with an extension ratio $\lambda_\parallel$) and deforms in the normal direction by the same extension ratio $\lambda_\perp$ as the central region. The position of a material point in the central region has the form

$$\mathbf{r}_{\mathrm{in}} = \lambda_{\mathrm{eq}}(x, y, \lambda_\perp z). \tag{13}$$

With Supplementary Equation (13), the trace and determinant of the metric tensor has the form $g_{\alpha\alpha} = \lambda_{\mathrm{eq}}^2(2 + \lambda_\perp^2)$ and $g = \lambda_{\mathrm{eq}}^6 \lambda_\perp^2$. The position of a material point in the surrounding region has the form

$$\mathbf{r}_{\mathrm{ex}} = \lambda_{\mathrm{eq}}(\lambda_\parallel x_{\mathrm{r}}, y_{\mathrm{r}}, \lambda_\perp z). \tag{14}$$

x_{r} and y_{r} are the coordinate systems in the radial and angular directions to the nanosheets, see Supplementary Figure 10**b**. With Supplementary Equation (14), the trace and determinant of the metric tensor has the form $g_{\alpha\alpha} = \lambda_{\mathrm{eq}}^2(1 + \lambda_\parallel^2 + \lambda_\perp^2)$ and $g = \lambda_{\mathrm{eq}}^6 \lambda_\perp^2 \lambda_\parallel^2$.

The volume density of the free energy of the central region has the form

$$f_{\text{gel}}^{\text{in}} = \frac{1}{2}G_0\left[\lambda_{\text{eq}}^2(2+\lambda_\perp^2)-3\right]+\frac{\phi_0}{\phi_{\text{in}}}f_{\text{sol}}(\phi_{\text{in}}). \tag{15}$$

The volume fraction ϕ_{in} of the polymer network in the central region has the form

$$\phi_{\text{in}} = \frac{\phi_{\text{eq}}}{\lambda_\perp}. \tag{16}$$

The volume density of the free energy of the surrounding region has the form

$$f_{\text{gel}}^{\text{ex}} = \frac{1}{2}G_0\left[\lambda_{\text{eq}}^2(1+\lambda_\parallel^2+\lambda_\perp^2)-3\right]+\frac{\phi_0}{\phi_{\text{ex}}}f_{\text{sol}}(\phi_{\text{ex}}). \tag{17}$$

The volume fraction ϕ_{ex} of the polymer network in the surrounding region has the form

$$\phi_{\text{ex}} = \frac{\phi_{\text{eq}}}{\lambda_\parallel\lambda_\perp}. \tag{18}$$

The free energy of the unit cell has the form

$$F_{\text{gel}} = \pi r_{\text{in}}^2 h_0 \frac{1}{\lambda_{\text{eq}}^3} f_{\text{gel}}^{\text{in}} + 2\pi(r_{\text{ex}}-r_{\text{in}})r_{\text{in}}h_0\frac{1}{\lambda_{\text{eq}}^3}f_{\text{gel}}^{\text{ex}}. \tag{19}$$

We treat a relatively short time scale, in which the volume of the gel is conserved. The conservation of the volume leads to the relationship

$$\pi r_{\text{in}}^2 h_0 + 2\pi(r_{\text{ex}}-r_{\text{in}})r_{\text{in}}h_0 = \pi r_{\text{in}}^2\lambda_\perp h_0 + 2\pi\lambda_\parallel(r_{\text{ex}}-r_{\text{in}})r_{\text{in}}\lambda_\perp h_0. \tag{20}$$

Supplementary Equation (20) is rewritten in the form

$$\lambda_\parallel = \frac{1}{2s}\left(\frac{1+2s}{\lambda_\perp}-1\right), \tag{21}$$

where the volume ratio s of the central and surrounding regions is defined by the form

$$s = \frac{r_{\rm ex} - r_{\rm in}}{r_{\rm in}}. \tag{22}$$

By substituting Supplementary Equation (21) into Supplementary Equation (19), the free energy $F_{\rm gel}$ is represented as a function only of the extension ratio $\lambda_\perp$ in the normal direction, see fig. 2 in the main article. Minimizing the free energy $F_{\rm gel}$ with respect to the extension ratio $\lambda_\perp$ leads to the force balance equation

$$-\frac{G_0}{\lambda_{\rm eq}}\left((1+2s)\lambda_\perp - \frac{(1+2s)^2}{2s}\frac{1}{\lambda_\perp^3} + \frac{1+2s}{2s}\frac{1}{\lambda_\perp^2}\right) + \Pi_{\rm sol}(\phi_{\rm in}) - \Pi_{\rm sol}(\phi_{\rm ex}) = 0. \tag{23}$$

Supplementary Note 3

Asymptotic solutions for small rescaled shear modulus

The extention ratio $\lambda_\perp$ and the volume fraction $\phi_{\rm in}$ are rewritten as functions of the volume fraction $\phi_{\rm ex}$;

$$\lambda_\perp = \frac{(1+2s)\phi_{\rm ex} - 2s\phi_{\rm eq}}{\phi_{\rm ex}} \tag{24}$$

$$\phi_{\rm in} = \frac{\phi_{\rm eq}\phi_{\rm ex}}{(1+2s)\phi_{\rm ex} - 2s\phi_{\rm eq}}, \tag{25}$$

where we used Supplementary Equations (16), (18), (21) to derive these relationships.

With Supplementary Equations (24) and (25), Supplementary Equation (23) provides the relationship between the volume fraction $\phi_{\rm ex}$ and the interaction parameter χ. We first represent the interaction parameter χ as a function of the volume fraction $\phi_{\rm ex}$. For small rescaled shear modulus g_0 (defined in Supplementary Equation (11)), the interaction

parameter has an asymptotic form

$$\chi \simeq g_0 \phi_{\rm eq}^{1/3} \frac{(1+2s)^2}{\phi_{\rm ex}^2} - \frac{\log(1-\phi_{\rm ex}) + \phi_{\rm ex}}{\phi_{\rm ex}^2}, \tag{26}$$

where we used Supplementary Equation (12) to derive this form. When the interaction parameter χ is large, the volume fraction $\phi_{\rm ex}$ has an asymptotic solution

$$\phi_{\rm ex} = 1 - {\rm e}^{-\chi-1}. \tag{27}$$

The volume fraction $\phi_{\rm ex}$ has an asymptotic form

$$\phi_{\rm ex} = 3\left(\chi - \frac{1}{2}\right) \tag{28}$$

near the interaction parameter $\chi_{\rm tr}$ at which the values of the free energy of the undeformed and deformed states are equal.

Supplementary Equation (27) implies that the volume fraction $\phi_{\rm ex}$ asymptotically approaches to unity for large values of the interaction parameter χ. In this asymptotic limit, the extention ratio $\lambda_\perp$ has the form

$$\lambda_\perp = 1 + 2s(1 - \phi_{\rm eq}), \tag{29}$$

where we used Supplementary Equation (24) to derive this form. Supplementary Equation (29) is equal to equation (2) in the main article. Supplementary Equation (29) is not an asymptotic form for small values of the rescaled shear modulus g_0 and is also effective to large values of the rescaled shear modulus g_0 (and even for cases in which the interaction parameter χ_0 of the solvent used for the swelling process is not zero).

The solution of the deformed state becomes unstable for $\chi < \chi_{\rm sp1}$. The volume fraction $\phi_{\rm ex}$ at the threshold value is derived by the condition $\partial\chi/\partial\phi_{\rm ex} = 0$. For small values of

the volume fraction $\phi_{\rm ex}$, the second term of Supplementary Equation (26) is expanded as a power series of $\phi_{\rm ex}$;

$$\chi = g_0 \phi_{\rm eq}^{1/3} \frac{(1+2s)^2}{\phi_{\rm ex}^2} + \frac{1}{2} + \frac{1}{3}\phi_{\rm ex}, \tag{30}$$

where higher order terms of $\phi_{\rm ex}$ are omitted. The volume fraction $\phi_{\rm ex}$ at the threshold value $\chi_{\rm sp1}$ thus has the form

$$\phi_{\rm ex} = (6 g_0 \phi_{\rm eq}^{1/3}(1+2s)^2)^{1/3}. \tag{31}$$

Substituting Supplementary Equation (31) into Supplementary Equation (30) leads to the form

$$\chi_{\rm sp1} = \frac{1}{2} + \frac{3}{6^{2/3}}(g_0 \phi_{\rm eq}^{1/3}(1+2s)^2)^{1/3}. \tag{32}$$

The values of the free energy of the undeformed and deformed states become equal for $\chi = \chi_{\rm tr}$. The free energy of the undeformed state has the form

$$\frac{F_{\rm gel}^{\rm und}}{\pi r_{\rm in}^2 h_0} = (1+2s)\frac{k_{\rm B}T}{v_0}\left[\frac{3}{2} g_0 \phi_{\rm eq}^{1/3} + \frac{v_0}{k_{\rm B}T} f_{\rm sol}(\phi_{\rm eq})\right], \tag{33}$$

where it is derived by substituting $\lambda_\perp = 1$ into Supplementary Equation (19). The free energy of the deformed state has the form

$$\frac{F_{\rm gel}^{\rm def}}{\pi r_{\rm in}^2 h_0} = \frac{k_{\rm B}T}{v_0}\left[\left(\frac{3}{2} + 4s + 6s^2 + 4s^3\right) g_0 \phi_{\rm eq}^{1/3} + (\chi - 1)\phi_{\rm eq} + 2s\frac{\phi_{\rm eq}}{\phi_{\rm ex}}\frac{v_0}{k_{\rm B}T} f_{\rm sol}(\phi_{\rm ex})\right], \tag{34}$$

where we derived this equation by substituting Supplementary Equations (21), (24), and (25) into Supplementary Equation (19) and by omitting higher order terms with respect to the rescaled shear modulus g_0 (see also Supplementary Equation (12)). The condition with which the values of the free energy of the deformed and undeformed states become equal

thus has the form

$$(1+6s+4s^2)g_0\phi_{\rm eq}^{1/3}+2\phi_{\rm eq}\left(1+\frac{1-\phi_{\rm ex}}{\phi_{\rm ex}}\log(1-\phi_{\rm ex})-\chi\phi_{\rm ex}\right)=0. \tag{35}$$

We expand the second term of Supplementary Equation (35) in a power series of the volume fraction $\phi_{\rm ex}$ and use Supplementary Equation (28) to derive the form

$$\chi_{\rm tr}=\frac{1}{2}+\sqrt{\frac{1}{3}(1+6s+4s^2)g_0\phi_{\rm eq}^{-2/3}}. \tag{36}$$

Substituting Supplementary Equation (12) into Supplementary Equation (36) leads to equation (3) in the main article.

The undeformed state becomes unstable for $\chi>\chi_{\rm sp2}$. To derive the threshold value, we set $\lambda_\perp=1+2s\epsilon$ and represent the free energy as a power series of ϵ;

$$\frac{F_{\rm gel}}{\pi r_{\rm in}^2 h_0}=\frac{F_{\rm gel}^{\rm und}}{\pi r_{\rm in}^2 h_0}+s(1+2s)\left[\frac{G_0}{\lambda_{\rm eq}}(1+8s)+\phi_{\rm eq}\Pi'_{\rm sol}(\phi_{\rm eq})\right]\epsilon^2, \tag{37}$$

where $\Pi'_{\rm sol}(\phi)$ is the derivative of the osmotic pressure $\Pi_{\rm sol}(\phi)$ with respect to the volume fraction ϕ. The undeformed state becomes unstable when the coefficient of ϵ^2 becomes negative. The threshold value $\chi_{\rm sp2}$ of the interaction parameter thus has the form

$$\chi_{\rm sp2}=\frac{1}{2}\frac{1}{1-\phi_{\rm eq}}+\frac{1}{2}g_0\phi_{\rm eq}^{-5/3}(1+8s). \tag{38}$$

Supplementary Equation (38) is indeed not the asymptotic form for small values of the rescaled shear modulus g_0 and thus is also effective for relatively large values of the modulus g_0 (and even for cases in which the interaction parameter χ_0 of the solvent used for the swelling process is not zero).

Supplementary Note 4

Lateral deformation of the central region

In the main article, we assumed that the central region of a unit cell does not deform in the lateral direction. One may think that the lateral deformation of the gel region is possible at far away from the nanosheets, see Supplementary Figure 1**a**. We here show that the lateral deformation is negligible for cases in which the height h_0 of the unit cell is smaller than the radius $r_{\rm in}$ of the nanosheets.

We treat a composite gel, which is prepared in a similar manner to the treatment in the main article: A polymer network is swollen in a solvent by the swelling ratio $\lambda_{\rm eq}$, see Supplementary Note 2. Solid nanosheets of radius $r_{\rm in}$ are then incorporated in the swollen gel. The solid nanosheets are cofacially oriented and are aligned in a stack of layers, which are parallel to the nanosheets. We treat the deformation of a unit cell when one increases the interaction parameter χ.

To take into account the non-uniform lateral deformation of the central region in a simple manner, we assume that the extension ratio $\lambda_{\rm in}(z)$ (of the central region) in the lateral direction is a function only of the distance z from the bottom nanosheet. The lateral extension ratio $\lambda_{\rm in}(z)$ has the form

$$\lambda_{\rm in}(z) = 1 + \epsilon_{\rm s}(z) \tag{39}$$

in the central region. $\epsilon_{\rm s}(z)$ is the strain that accounts for the non-uniform lateral deformation with $\epsilon_{\rm s}(h_0/\lambda_{\rm eq}) = \epsilon_{\rm s}(0) = 0$ due to the restraint of the nanosheets. With this treatment, the position $\mathbf{r}_{\rm in}$ of the material point in the central region after the deformation thus has the form

$$\mathbf{r}_{\rm in} = \lambda_{\rm eq}(\lambda_{\rm in}(z)x, \lambda_{\rm in}(z)y, \lambda_{\perp}z). \tag{40}$$

For simplicity, we assume that the extension ratio $\lambda_\perp$ in the normal to the nanosheet is uniform. Supplementary Equation (40) returns to equation (10) in the main article for $\epsilon_{\rm s}(z) = 0$ (see also Supplementary Equation (13)).

The position $\mathbf{r}_{\rm ex}$ of the material point in the surrounding region after the deformation thus has the form

$$\mathbf{r}_{\rm ex} = \lambda_{\rm eq}(\lambda_{\rm ex}(z) x_{\rm r}, y_{\rm r}, \lambda_\perp z), \tag{41}$$

where $\lambda_{\rm ex}(z)$ is the extension ratio (of the surrounding region) in the lateral direction. The unit cells are cylindrical even with the non-uniform deformation $\epsilon_{\rm s}(z)$ because of the continuity of the gel and thus $\lambda_{\rm in}(z) r_{\rm in} + \lambda_{\rm ex}(z)(r_{\rm ex} - r_{\rm in})$ is constant. The extension ratio $\lambda_{\rm ex}(z)$ thus has the form

$$\lambda_{\rm ex}(z) = \lambda_{\rm s\|} - \frac{\epsilon_{\rm s}(z)}{s}, \tag{42}$$

where s is the volume ratio of the central and surrounding regions (see equation (13) in the main article and Supplementary Equation (22)). The extension ratio $\lambda_{\rm ex}(z)$ returns to a constant $\lambda_{\rm s\|}$ for $\epsilon_{\rm s}(z) = 0$.

In a time scale, before the solvent permeates out from the gel, the volume of the unit cell is constant during the deformation. The parameter $\lambda_{\rm s\|}$ thus depends on the extension ratio $\lambda_\perp$ and is derived by using the form

$$\frac{1+2s}{\lambda_\perp} = \frac{\lambda_{\rm eq}}{h_0} \int_0^{h_0/\lambda_{\rm eq}} dz \, \left(1 + 2s\lambda_{\rm s\|} + 2s\lambda_{\rm s\|}\epsilon_{\rm s}(z) - \epsilon_{\rm s}^2(z)\right). \tag{43}$$

Supplementary Equation (43) returns to Supplementary Equation (21) for $\epsilon_{\rm s}(z) = 0$.

The free energy of the unit cell is derived by using the Supplementary Equation (40) and (41) (see Supplementary Methods for the derivation). The free energy density in the central

region has the form

$$f_{\rm gel}^{\rm in} \;=\; \frac{1}{2}G_0\left[\lambda_{\rm eq}^2\left(2\lambda_{\rm in}^2(z)+\lambda_\perp^2+\lambda_{\rm in}'^2(z)r^2\right)-3\right]+\frac{\phi_0}{\phi_{\rm in}}f_{\rm sol}(\phi_{\rm in}), \tag{44}$$

where G_0 is the shear modulus of the polymer network and $f_{\rm sol}(\phi)$ is the mixing free energy of the gel, see Supplementary Equation (6). r $(=\sqrt{x^2+y^2})$ is the distance from the central axis of the unit cell. The free energy density in the surrounding region has the form

$$f_{\rm gel}^{\rm ex} \;=\; \frac{1}{2}G_0\left[\lambda_{\rm eq}^2\left(1+\lambda_\perp^2+\lambda_{\rm ex}^2(z)+\lambda_{\rm ex}'^2(z)x_{\rm r}^2\right)-3\right]+\frac{\phi_0}{\phi_{\rm ex}}f_{\rm sol}(\phi_{\rm ex}). \tag{45}$$

The free energy of the unit cell thus has the form

$$F_{\rm gel} \;=\; \int_0^{r_{\rm in}/\lambda_{\rm eq}} dr\,2\pi r\int_0^{h_0/\lambda_{\rm eq}} dz\,f_{\rm gel}^{\rm in}+2\pi r_{\rm in}\int_0^{(r_{\rm ex}-r_{\rm in})/\lambda_{\rm eq}} dx_{\rm r}\int_0^{h_0/\lambda_{\rm eq}} dz\,f_{\rm gel}^{\rm ex}. \tag{46}$$

The free energy $F_{\rm gel}$ is a functional of the extension ratio $\lambda_\perp$ and the strain $\epsilon_{\rm s}(z)$. The third term in the round bracket of Supplementary Equation (44) and the fourth term in the round bracket of Supplementary Equation (45) are the elastic energy due to the shear deformation and both scale as $r_{\rm in}^2\epsilon_{\rm m}^2/h_0^2$ with the aspect ratio $r_{\rm in}/h_0$ of the unit cell. These elastic energy contributions suppress the non-uniform lateral deformation of the central region. The mixing free energy does not depend on the aspect ratio $r_{\rm in}/h_0$.

For cases in which the height h_0 of the unit cell is smaller than the radius $r_{\rm in}$ of the nanosheets, it is reasonable to assume that the strain $\epsilon_{\rm s}(z)$ has an approximate form

$$\epsilon_{\rm s}(z)=4\epsilon_{\rm m}\frac{\lambda_{\rm eq}^2}{h_0^2}z\left(\frac{h_0}{\lambda_{\rm eq}}-z\right), \tag{47}$$

which are leading order terms in a power series with respect to the distance z. $\epsilon_{\rm m}$ is the strain at the middle plane $z=h_0/(2\lambda_{\rm eq})$. By substituting Supplementary Equation (47) into Supplementary Equation (46), the free energy $F_{\rm gel}$ is derived as a function of the strain $\epsilon_{\rm m}$

and the extension ratio $\lambda_\perp$. We derive the strain $\epsilon_{\rm m}$ and the extension ratio $\lambda_\perp$ as a function of the interaction parameter χ by minimizing the free energy.

For cases in which a parameter $\epsilon_{\rm m}/(s\lambda_{\rm s\|})$ is small, the free energy $F_{\rm gel}$ has an approximate form

$$F_{\rm gel}(\lambda_\perp, \epsilon_{\rm m}) \simeq F_{\rm gel}(\lambda_\perp, \epsilon_{\rm m} = 0) + \Lambda_1 \epsilon_{\rm m} + \frac{1}{2}\Lambda_2 \epsilon_{\rm m}^2, \tag{48}$$

which is derived by expanding Supplementary Equation (46) in a power series of $\epsilon_{\rm m}$ and then by omitting higher order terms. The coefficients, Λ_1 and Λ_2, have the forms

$$\begin{aligned}
\Lambda_1 &= \frac{4}{3}\frac{G_0}{\lambda_{\rm eq}}\left(1 + \frac{1}{4s} - \frac{(1+2s)^2}{4s}\frac{1}{\lambda_\perp^2}\right) \\
&\quad -\frac{4}{3}\lambda_\perp \Pi_{\rm sol}(\phi_{\rm in}^0) + \frac{2}{3}\left(\frac{1+2s}{\lambda_\perp} + 1\right)\lambda_\perp \Pi_{\rm sol}(\phi_{\rm ex}^0) \qquad (49) \\
\Lambda_2 &= \frac{8}{3}\frac{G_0}{\lambda_{\rm eq}}\frac{r_{\rm in}^2}{h_0^2}\left(1 + \frac{4}{3}s\right) + \frac{8}{3}\frac{G_0}{\lambda_{\rm eq}}\left(s\lambda_\|^2 + \frac{16}{15}\lambda_\| + \frac{2}{5}\frac{1+s}{s}\right) \\
&\quad +\frac{16}{5}\frac{\phi_{\rm eq}}{\phi_{\rm in}^0}\Pi_{\rm sol}(\phi_{\rm in}^0) + \frac{32}{15}\frac{\phi_{\rm eq}}{\phi_{\rm in}^0}\left(\phi_{\rm in}^0 \Pi'_{\rm sol}(\phi_{\rm in}^0) - 2\Pi_{\rm sol}(\phi_{\rm in}^0)\right) \\
&\quad +\frac{16}{15}\frac{1}{\lambda_\|}\left(\frac{7}{3} + \frac{2}{s\lambda_\|}\right)\frac{\phi_{\rm eq}}{\phi_{\rm ex}^0}\Pi_{\rm sol}(\phi_{\rm ex}^0) \\
&\quad +\frac{8}{3}\left(\frac{s}{3} + \frac{2}{3}\frac{1}{\lambda_\|} + \frac{2}{5}\frac{1}{s\lambda_\|^2}\right)\frac{\phi_{\rm eq}}{\phi_{\rm ex}^0}\left(\phi_{\rm ex}^0 \Pi'_{\rm sol}(\phi_{\rm ex}^0) - 2\Pi_{\rm sol}(\phi_{\rm ex}^0)\right). \qquad (50)
\end{aligned}$$

The lateral extension ratio $\lambda_\|$ is defined by Supplementary Equation (21). $\phi_{\rm in}^0$ ($\equiv \phi_{\rm eq}/\lambda_\perp$) and $\phi_{\rm ex}^0$ ($\equiv \phi_{\rm eq}/(\lambda_\perp \lambda_\|)$) are the volume fractions of the polymer network in the central and surrounding regions for $\epsilon_{\rm m} = 0$, respectively.

Minimizing the free energy $F_{\rm gel}$ with respect to the strain $\epsilon_{\rm m}$ leads to the form

$$\epsilon_{\rm m} = -\frac{\Lambda_1}{\Lambda_2}, \tag{51}$$

see Supplementary Equation (48). The strain $\epsilon_{\rm m}$ thus has an asymptotic form

$$\epsilon_{\rm m} \simeq \frac{s}{1+4s/3}\frac{h_0^2}{r_{\rm in}^2}\frac{\phi_{\rm eq}^{5/3}}{2g_0}\chi \tag{52}$$

for $\lambda_\perp = 1$ and large values of the aspect ratio $r_{\rm in}/h_0$. The strain $\epsilon_{\rm m}$ thus increases linearly with increasing the interaction parameter χ in the undeformed state, $\lambda_\perp = 1$, see Supplementary Figure 1**b**. In the deformed state, the strain $\epsilon_{\rm m}$ shows a maximum value $\epsilon_{\rm m,max}$ and then decreases with increasing the interaction parameter χ for large values of the interaction parameter χ. The maximum value $\epsilon_{\rm m,max}$ decreases with increasing the aspect ratio $r_{\rm in}/h_0$ of the unit cell, see Supplementary Figure 1**c**. More quantitatively, the strain $\epsilon_{\rm m,max}$ indeed scales as $(r_{\rm in}/h_0)^{-2}$ for cases in which the aspect ratio $r_{\rm in}/h_0$ is large enough, see Supplementary Figure 1**d**; Γ_1 does not depend on the aspect ratio $r_{\rm in}/h_0$ of the unit cell and Γ_2 has a term that scales as $r_{\rm in}^2/h_0^2$ (see the first term of Supplementary Equation (50)). We thus conclude that the lateral deformation of the central region is suppressed by the elastic energy and is negligible for cases in which the aspect ratio $r_{\rm in}/h_0$ is large, see also Supplementary Figure 2.

Supplemenatry Note 5

Dynamics of the deformation of the composite gel

We here derive the time scale of the deformation of the composite gel by using the Onsager principle.[1,2] The time evolution equation of the gel dynamics has been derived by using this principle.[1,2] For simplicity, we treat cases in which the thickness $r_{\rm ex} - r_{\rm in}$ of the surrounding region is much smaller than the radius $r_{\rm in}$ of the nanosheets so that the surrounding region is in the local equilibrium. In these cases, the deformation of the composite gel is represented

by the displacement vector $\mathbf{u}(\mathbf{r})$ of the central region

$$\mathbf{u}(\mathbf{r}) = (0, 0, \epsilon(t)z_{\rm s}), \tag{53}$$

where $\epsilon(t)$ is the strain in the normal direction and we use the cylindrical coordinate system $\mathbf{r}_{\rm s} = (x_{\rm s}, y_{\rm s}, z_{\rm s})$ for the swollen gel; $x_{\rm s}$ and $y_{\rm s}$ are the coordinates in the parallel to the nanosheets and $z_{\rm s}$ is the distance from the nanosheet at the bottom of the unit cell, see the Supplementary Figure 10**c**. The strain $\epsilon(t)$ of the central region has a relationship

$$\epsilon(t) = \lambda_{\perp}(t) - 1 \tag{54}$$

with the extension ratio $\lambda_{\perp}$ in the normal direction. We derive the strain $\epsilon(t)$ as a function of time t by using the lubrication approximation, which is effective for cases in which the distance h_0 between the registered nanosheets is smaller than the radius $r_{\rm in}$ of the nanosheets.

The Onsager principle states that the time evoluation equation is derived by minimizing the Rayleighan that has the form

$$\mathcal{R} = \Phi + \dot{F}_{\rm gel} + \mathcal{R}_{\rm con}, \tag{55}$$

where Φ is the dissipation function, $\dot{F}_{\rm gel}$ is the time derivative (indicated by the dot) of the free energy of the unit cell, and $\mathcal{R}_{\rm con}$ is the Lagrange multiplier that ensures that the space is occupied by either solvent molecules or monomers. Here and after, the dot above a physical quantity represents the time derivative of the quantity.

The dissipation function Φ has the form

$$\Phi = \frac{1}{2}\int_{V_{\rm in}} dV\, \xi(\dot{\mathbf{u}}(\mathbf{r}) - \mathbf{v}_{\rm sol}(\mathbf{r}))^2, \tag{56}$$

where $\dot{\mathbf{u}}(\mathbf{r})$ is the time derivative of the displacement vector $\mathbf{u}(\mathbf{r})$ and $\mathbf{v}_{\rm sol}(\mathbf{r})$ is the velocity

field of the solvent. ξ is the constant that accounts for the friction between the solvent and the polymer network. F_{gel} is the time derivative of the free energy of the gel, see also Supplementary Equation (19). The Lagrange multiplier has the form

$$\mathcal{R}_{\mathrm{con}} = -\int_{V_{\mathrm{in}}} dV\, p(\mathbf{r}) \nabla \cdot (\phi \dot{\mathbf{u}}(\mathbf{r}) + (1-\phi)\mathbf{v}_{\mathrm{sol}}(\mathbf{r})), \tag{57}$$

where $p(\mathbf{r})$ is the hydrostatic pressure and ϕ is the volume fraction of the polymer network. Because the thickness of the surrounding region is very small, solvent molecules travel the central region most of the time. We thus neglected the dissipation function and the Lagrange multiplier in the surrounding region and ensure that the sum of the volumes of the central and surrounding regions is constant by using Supplementary Equation (21).

The Rayleighan is a functional of the strain $\epsilon(t)$ and the velocity field $\mathbf{v}_{\mathrm{sol}}(\mathbf{r})$ of the solvent. Minimizing the Rayleighan with respect to the velocity field $\mathbf{v}_{\mathrm{sol}}(\mathbf{r})$ leads to the Darcy's law that has the form

$$(1-\phi)(\mathbf{v}_{\mathrm{sol}}(\mathbf{r}) - \dot{\mathbf{u}}(\mathbf{r})) = -\kappa \nabla p(\mathbf{r}), \tag{58}$$

where κ ($\equiv (1-\phi)^2/\xi$) is the Darcy's constant. The relationship

$$\nabla \cdot (\phi \dot{\mathbf{u}}(\mathbf{r}) + (1-\phi)\mathbf{v}_{\mathrm{sol}}(\mathbf{r})) = 0, \tag{59}$$

accounts for the fact that the space is occupied by either solvent molecules or monomers, see Supplementary Equation (57). Substituting Supplementary Equation (58) into Supplementary Equation (59) leads to the form

$$\kappa \frac{1}{r_{\mathrm{s}}} \frac{\partial}{\partial r_{\mathrm{s}}} \left(r_{\mathrm{s}} \frac{\partial}{\partial r_{\mathrm{s}}} p(r_{\mathrm{s}}) \right) = \dot{\epsilon}(t). \tag{60}$$

To derive Supplementary Equation (60), we used the lubrication approximation, which as-

sumes that the hydrostatic pressure $p(r_{\mathrm{s}})$ does not depend on z_{s} (which is the distance from the bottom nanosheet). r_{s} is the distance from the z_{s}-axis.

The hydrostatic pressure $p(r_{\mathrm{s}})$ is derived in the form

$$p(r_{\mathrm{s}}) = p_{\mathrm{ex}} + \frac{1}{4}\frac{\dot{\epsilon}}{\kappa}(r_{\mathrm{s}}^2 - r_{\mathrm{in}}^2) \tag{61}$$

by solving Supplementary Equation (60), where we used the boundary condition $p_{\mathrm{ex}} = p(r_{\mathrm{in}})$ (and the fact that the hydrostatic pressure does not diverge for $r \to 0$) to derive this equation. The velocity field $\mathbf{v}_{\mathrm{sol}}(\mathbf{r})$ thus has the form

$$(1-\phi)(\mathbf{v}_{\mathrm{sol}}(\mathbf{r}) - \dot{\mathbf{u}}(\mathbf{r})) = -\frac{1}{2}\dot{\epsilon} r_{\mathrm{s}} \mathbf{e}_r, \tag{62}$$

where $\mathbf{e}_r$ is the unit vector in the radial direction. Substituting Supplementary Equation (62) into Supplementary Equation (55) leads to the form

$$\mathcal{R} = \frac{1}{16}\frac{r_{\mathrm{in}}^2}{\kappa}\pi r_{\mathrm{in}}^2 h_0 \dot{\epsilon}^2(t) + \dot{\epsilon}(t)\frac{\partial}{\partial \lambda_\perp} F_{\mathrm{gel}}. \tag{63}$$

Minimizing Supplementary Equation (63) with respect to $\dot{\epsilon}(t)$ leads to the form

$$\tau_0 \frac{d}{dt}\epsilon(t) = -\frac{1}{K_{\mathrm{i}} + 4G_{\mathrm{i}}/3}\frac{\partial}{\partial \lambda_\perp}\left(\frac{F_{\mathrm{gel}}}{\pi r_{\mathrm{in}}^2 h_0}\right), \tag{64}$$

where τ_0 is the time scale with which solvent molecules travel by the distance r_{in} and has the form

$$\tau_0 = \frac{r_{\mathrm{in}}^2}{8\kappa(K_{\mathrm{i}} + 4G_{\mathrm{i}}/3)}, \tag{65}$$

see also eqs. (78) and (146) in ref.[2] The constant $K_{\mathrm{i}} + 4G_{\mathrm{i}}/3$ is the elastic modulus of the

gel before the interaction parameter χ is changed and has the form

$$K_{\rm i} + \frac{4}{3}G_{\rm i} = \frac{k_{\rm B}T}{v_0}\left(g_0\phi_{\rm eq}^{1/3} + \frac{\phi_{\rm eq}^2}{1-\phi_{\rm eq}}\right). \tag{66}$$

Supplementary Equation (64) is analogous to the equation of motion of model A, which is driven by the thermal fluctuations.[3] Indeed, the derivative of the free energy $\partial F_{\rm gel}/\partial\lambda_\perp$ is zero at the initial state $\epsilon(t) = 0$ and thus the thermal fluctuation is necessary to drive the deformation of the composite gel. To simplify the notation, we introduce a function

$$\begin{aligned}\Gamma(\epsilon) &= \frac{1}{K_{\rm i}+4G_{\rm i}/3}\frac{\partial}{\partial\lambda_\perp}\left(\frac{F_{\rm gel}}{\pi r_{\rm in}^2 h_0}\right) \\ &\simeq \frac{1}{K_{\rm i}+4G_{\rm i}/3}\left[A\epsilon + B + \frac{v_0}{k_{\rm B}T}\Pi_{\rm sol}\left(\phi_{\rm ex}\right)\right],\end{aligned} \tag{67}$$

which equal to the right-hand-side of Supplementary Equation (64). In the last form of Supplementary Equation (67), we used paramters

$$A = g_0\phi_{\rm eq}^{1/3}\frac{(1+4s)^2}{2s} + g_0\phi_{\rm eq}^{1/3} + \frac{\phi_{\rm eq}^2}{1-\phi_{\rm eq}} - 2\chi\phi_{\rm eq}^2 \tag{68}$$

$$B = \chi\phi_{\rm eq}^2 - g_0\phi_{\rm eq}^{1/3} \tag{69}$$

and the relationship $\phi_{\rm ex} = 2s\phi_{\rm eq}/(2s-\epsilon)$.

To analyze the dynamics of the gel in the short time scale, we expand the right-hand-side of Supplementary Equation (64) in a power series of $\epsilon(t)$ and omit higher order terms. This leads to the form

$$\tau_0\frac{d}{dt}\epsilon(t) \simeq \alpha_0\epsilon(t), \tag{70}$$

where the dimensionless parameter α_0 has the form

$$\alpha_0^{-1} = \frac{1}{2\phi_{\rm eq}^2(1+2s)}\frac{K_{\rm i}+4G_{\rm i}/3}{\chi-\chi_{\rm sp2}}, \tag{71}$$

see Supplementary Figure 6. We used Supplementary Equation (38) to derive this expression. The strain $\epsilon(t)$ thus has an asymptotic form

$$\epsilon(t) = \epsilon_0 \mathrm{e}^{\alpha_0 t/\tau_0}, \tag{72}$$

where ϵ_0 is the strain at $t = 0$ and is due to the thermal fluctuation. The dimensionless parameter α_0 has an asymptotic form

$$\alpha_0^{-1} = \frac{3}{2}\frac{s}{1+2s}\frac{1}{\chi - \chi_{\mathrm{sp2}}} \tag{73}$$

when the rescaled shear modulus g_0 is small.

The strain ϵ_∞ in the deformed state ($t \to \infty$) is derived by using the force balance equation $\partial F_{\mathrm{gel}}/\partial \lambda_\perp = 0$. To analyze the dynamics in the long time scale, we expand the right-hand-side of Supplementary Equation (64) in the power series of $\epsilon(t) - \epsilon_\infty$. This leads to the form

$$\tau_0 \frac{d}{dt}\epsilon(t) \simeq -\alpha_\infty(\epsilon(t) - \epsilon_\infty), \tag{74}$$

where the dimensionless parameter α_∞ has the form $\alpha_\infty \equiv -\Gamma'(\epsilon_\infty)$ ($\Gamma'(\epsilon)$ is the derivative of $\Gamma(\epsilon)$ with respect to ϵ). The strain $\epsilon(t)$ thus has an asymptotic form

$$\epsilon(t) = \epsilon_\infty \left(1 - \mathrm{e}^{-\alpha_\infty t/\tau_0}\right), \tag{75}$$

see Supplementary Figure 11.

Supplementary Note 6

Possible effects of slip between the nanosheets and the polymer network

Even for cases in which the adhesion between the nanosheets and the polymer network is not large enough, the polymer network in the surrounding region is not likely to slide into the central region because the entire region of the gel is connected by the polymer network and the elastic energy generated by the shear deformation of the polymer network suppresses the lateral deformation of the central region. At a first glance, one may think about another possibility that when the slip between the polymer network and the nanosheets is allowed, the polymer network in the central region may deform in the lateral direction non-uniformly to locally decrease the free energy. With this deformation, the volume of the central region does not change because the polymer network in the surrounding region does not slide into the central region. We here use a simple argument to show that such a non-uniform deformation does not happen as long as the polymer network does not show phase separation even without the embedded nanosheets.

We here simplify the non-uniform deformation of the polymer network in the central region as follows: the cylindrical region $r < r_{\rm sep}/\lambda_{\rm eq}$ deforms uniformly with an extension ratio λ_1 (region 1) and the other region of the central region $r_{\rm sep}/\lambda_{\rm eq} < r < r_{\rm in}/\lambda_{\rm eq}$ deforms uniformly with the extension ratio λ_2 (region 2). In a more precise treatment, one must take into account the fact that the extension ratio in the direction parallel to the interface between region 1 and 2 is continuous across the interface. For simplicity, we here neglect the interfacial energy due to this continuity condition. With this treatment, the free energy of the central region has the form

$$F_{\rm gel}^{\rm in} = \frac{\pi r_{\rm sep}^2 h_0}{\lambda_{\rm eq}^3} f_{\rm gel}^{\rm sep}(\lambda_1) + \pi h_0 \frac{r_{\rm in}^2 - r_{\rm sep}^2}{\lambda_{\rm eq}^3} f_{\rm gel}^{\rm sep}(\lambda_2) \tag{76}$$

$$+\Pi_{\rm sep}\left[\pi r_{\rm sep}^2 \lambda_1^2 + \lambda_2^2(\pi r_{\rm in}^2 - \pi r_{\rm sep}^2) - \pi r_{\rm in}^2\right] h_0 \lambda_\perp, \tag{77}$$

where $\Pi_{\rm sep}$ is the Lagrange multiplier to ensure that the volume of the central region is constant. The first term of Supplementary Equation (77) is the free energy of the region 1 and the second term is the free energy of the region 2. The free energy density $f_{\rm gel}^{\rm sep}(\lambda)$ has the form

$$f_{\rm gel}^{\rm sep}(\lambda) = \frac{1}{2}G_0(\lambda_{\rm eq}^2(2\lambda^2 + \lambda_\perp^2) - 3) + \frac{\phi_0}{\phi}f_{\rm sol}(\phi), \tag{78}$$

where ϕ is the volume fraction of the polymer network and has the form

$$\phi = \frac{\phi_0}{\lambda^2\lambda_\perp}. \tag{79}$$

Supplementary Equation (78) is derived by assuming that the polymer network deforms uniformly in the lateral direction with an extension ratio λ (see the Supplementary Methods).

The extension ratio, λ_1 and λ_2, and the position $r_{\rm sep}$ of the interface are derived by minimizing the free energy $F_{\rm gel}^{\rm in}$ with respect to λ_1, λ_2, and $r_{\rm sep}$. Minimizing the free energy with respect to the extension ratios, λ_1 and λ_2, leads to the form

$$\Pi_{\rm sep} = \Pi_{||}(\lambda_1) = \Pi_{||}(\lambda_2), \tag{80}$$

where the function $\Pi_{||}(\lambda)$ has the form

$$\begin{aligned} \Pi_{||}(\lambda) &= -\frac{1}{\lambda\lambda_\perp\lambda_{\rm eq}^3}\frac{\partial}{\partial\lambda}f_{\rm gel}^{\rm sep}(\lambda) \\ &= -\frac{G_0}{\lambda_\perp\lambda_{\rm eq}} + \Pi_{\rm sol}(\phi). \end{aligned} \tag{81}$$

Supplementary Equation (80) indeed represents the continuity of the force (in the normal to the interface) across the interface. Minimizing the free energy with respect to the position

r_{sep} has the form

$$\mu_{\mathrm{sep}}(\lambda_1) = \mu_{\mathrm{sep}}(\lambda_2), \tag{82}$$

where the function $\mu_{\mathrm{sep}}(\lambda)$ has the form

$$\begin{aligned} \mu_{\mathrm{sep}}(\lambda) &= f_{\mathrm{gel}}^{\mathrm{sep}}(\lambda) - \frac{1}{2}\lambda\frac{\partial}{\partial\lambda}f_{\mathrm{gel}}^{\mathrm{sep}}(\lambda) \\ &= \frac{\partial}{\partial\phi}(\phi f_{\mathrm{gel}}^{\mathrm{sep}}(\lambda)). \end{aligned} \tag{83}$$

We used Supplementary Equations (80) and (81) to derive Supplementary Equation (82). Supplementary Equation (82) indeed represents the equality of chemical potentials in region 1 and 2. Supplementary Equations (80) and (82) imply that the polymer network in the central region is divided into two regions when both of the functions $\Pi_{\|}(\lambda)$ and $\mu(\lambda)$ to have, at least, two stable solutions (where $\partial\Pi_{\|}(\lambda)/\partial\phi > 0$ and $\partial\mu(\lambda)/\partial\phi > 0$). For cases in which the osmotic pressure has the form of Supplementary Equation (10), the functions, $\Pi_{\|}(\lambda)$ and $\mu(\lambda)$, only have one stable solution.

Supplementary Discussion

When the interaction parameter χ is changed relatively fast, an undeformed gel changes to the deformed state at the second threshold value χ_{sp2} and a deformed gel changes to the undeformed state at the first threshold value χ_{sp1}, see fig. 3 in the main article. These threshold values increase with increasing the volume ratio s and with increasing the rescaled shear modulus g_0 (see Supplementary Figure 7). The threshold values have asymptotic forms for small rescaled shear modulus g_0 (see Supplementary Equations (32) and (38)).

Our theory predicts that the composite gel shows a critical point, where the two threshold values become equal, at a relatively large value $g_{0\mathrm{c}}(> 0.27)$ of the rescaled shear modulus, see Supplementary Figure 8. For larger values of the rescaled shear modulus g_0, the composite

gel shows a discontinuous deformation in the reverse direction; the composite gel shrinks in the normal direction and extends in the radial direction. The composite gel deforms only moderately due to the large rescaled shear modulus g_0. The free energy decrease due to the shrinking of the central region (in the normal direction) dominates the free energy increase due to the extension of the surrounding region (in the radial direction) because the volume of the central region is larger than the volume of the surrounding region. The critical value of the rescaled shear modulus (which corresponds to about 1 crosslink per 4 monomers) is rather large and thus may not be experimentally relevant.

Supplementary References

(1) Doi, M. *Soft Matter Physics* (Oxford Univ. Press, 2013).

(2) Doi, M. Gel Dynamics. *J. Phys. Soc. Jpn.* **78,** 052001 (2009).

(3) Chaikin, P. M. & Lubensky, T. C. *Principle of condensed matter physics* (Cambridge Univ. Press, 1995).
